# May the successor of a singular cardinal be Jónsson?

**DOI:** 10.1007/s40574-025-00525-z

**Published:** 2026-01-07

**Authors:** Assaf Rinot

**Affiliations:** Department of Mathematics, Bar-Ilan University, 5290002 Ramat Gan, Israel

**Keywords:** Jónsson cardinal, Singular cardinals combinatorics, Club-guessing, PCF theory, Primary 03E02, Secondary 03E35, 03E55

## Abstract

Whether the successor of a singular cardinal can be Jónsson is a very old and famous open problem in set theory. Here, we collect necessary conditions for an affirmative answer, and put forward a list of closely-related questions in singular cardinals combinatorics that could eventually lead to settling the main problem at hand.

## Introduction

Our starting point is Ramsey’s theorem [41] asserting that for every 2-coloring of the unordered pairs of an infinite set *X*,  $c:[X]^2 \rightarrow 2,$ there exists an infinite subset Y⊆X that is *c-homogeneous* meaning that *c* is constant over $[Y]^2.$ Soon after, Sierpiński [63] demonstrated that Ramsey’s theorem does not generalize to the uncountable by constructing a coloring $c:[{\mathbb {R}}]^2\rightarrow 2$ satisfying that every uncountable $Y\subseteq {\mathbb {R}}$ is *c-omnichromatic*, meaning that $c\mathbin \upharpoonright [Y]^2$ is a surjection.

Instead of interpreting Sierpiński’s result as a disappointment, set theorists view witnesses to the failure of generalized Ramsey theorem as *strong colorings* and set their goal to make them as strong as possible. A succinct way to state results of this form is using the following *arrow notation*:

### Definition 1.1

[11, §18] For cardinals $\kappa ,\theta ,\lambda$ and a positive integer *n*:

- $\kappa \nrightarrow [\lambda ]^n_\theta$ asserts the existence of a coloring $c:[\kappa ]^n\rightarrow \theta$ such that $c\mathbin \upharpoonright [Y]^n$ is surjective for every $Y\subseteq \kappa$ of size λ;
- $\kappa \nrightarrow [\lambda ]^{<\omega }_\theta$ asserts the existence of a coloring $c:[\kappa ]^{<\omega }\rightarrow \theta$ such that $c\mathbin \upharpoonright [Y]^{<\omega }$ is surjective for every $Y\subseteq \kappa$ of size λ.

An infinite cardinal κ is *Jónsson* iff $\kappa \rightarrow [\kappa ]^{<\omega }_\kappa$ holds, i.e., $\kappa \nrightarrow [\kappa ]^{<\omega }_\kappa$ fails. Keisler and Rowbottom [30] proved that Jónsson cardinals do not exist in Gödel’s constructible universe. Rowbottom [48] proved that every measurable cardinal is a (regular) Jónsson cardinal, and Prikry [39] proved that a measurable cardinal may be turned into a singular Jónsson cardinal via a cardinal-preserving forcing. Tryba [68] and independently Woodin proved that if κ is a Jónsson cardinal then every stationary subset of κ reflects. This was then improved by Todorčević [67] who derived the same conclusion from $\kappa \rightarrow [\kappa ]^2_\kappa .$ In particular, the successor of a regular cardinal is never Jónsson. This survey centers around the open problem of whether the successor of a singular cardinal may consistently be Jónsson.

### Notation and conventions

Throughout this paper, κ denotes a regular uncountable cardinal, λ and μ denote infinite cardinals, and θ a (possibly finite) cardinal. We identify $[\kappa ]^2$ with $\{ (\alpha ,\beta )\mid \alpha<\beta <\kappa \},$ and likewise identify $[\kappa ]^{<\omega }$ with the collection of all finite increasing sequences of ordinals in κ. We let $E^\kappa _\mu :=\{\alpha < \kappa \mid {{\,\textrm{cf}\,}}(\alpha ) = \mu \},$ and define $E^\kappa _{\le \mu },$ $E^\kappa _{<\mu },$ $E^\kappa _{\ge \mu },$ $E^\kappa _{>\mu },$ $E^\kappa _{\ne \mu }$ analogously. ${{\,\textrm{Reg}\,}}(\kappa )$ stands for the set of all infinite regular cardinals below κ. For a set of ordinals *A*,  we write ${{\,\textrm{acc}\,}}(A) := \{\alpha \in A \mid \sup (A \cap \alpha ) = \alpha > 0\}$ and ${{\,\textrm{nacc}\,}}(A) := A {\setminus } {{\,\textrm{acc}\,}}(A).$

## Cardinal arithmetic

### Theorem 2.1

(Erdős–Hajnal–Rado, [11]) Suppose that λ is an infinite cardinal satisfying that $2^\lambda =\lambda ^+.$ Then $\lambda ^+\nrightarrow [\lambda ^+]^2_{\lambda ^+}$ holds.

### Proof

Using $2^\lambda =\lambda ^+,$ let $\langle a_\alpha \mid \alpha <\lambda ^+\rangle$ enumerate all bounded subsets of $\lambda ^+.$ We shall need the following standard claim concerning disjoint refinements.

### Claim 2.1.1

Let $\beta <\lambda ^+.$ There exists a pairwise disjoint subfamily ${\mathcal {B}}_\beta \subseteq [\beta ]^\lambda$ satisfying that for every $\alpha <\beta$ such that $a_\alpha \in [\beta ]^\lambda ,$ there is $b\in {\mathcal {B}}_\beta$ with $b\subseteq a_\alpha .$

### Proof

To avoid trivialities, suppose that $A_\beta :=\{\alpha <\beta \mid a_\alpha \in [\beta ]^\lambda \}$ is nonempty, so that $0<|A_\beta |\le \lambda .$ Fix a bijection $\pi :\lambda \leftrightarrow \lambda \times A_\beta$ and then recursively construct an injection $f:\lambda \rightarrow \beta$ satisfying that for every i<λ:

$$\begin{aligned} (\pi (i)=(j,\alpha ))\implies (f(i)\in a_\alpha ). \end{aligned}$$

For every $\alpha \in A_\beta ,$ the set $b_\alpha :=\{ f(i)\mid \exists j\,(\pi (i)=(j,\alpha ))\}$ is a λ-sized subset of $a_\alpha ,$ and since *f* is injective, ${\mathcal {B}}_\beta :=\{ b_\alpha \mid \alpha \in A_\beta \}$ is a pairwise disjoint family. □

Let $\langle {\mathcal {B}}_\beta \mid \beta <\lambda ^+\rangle$ be given by the preceding claim. Pick a coloring $c:[\lambda ^+]^2\rightarrow \lambda ^+$ satisfying that for every $\beta <\lambda ^+$ for every $b\in {\mathcal {B}}_\beta ,$ $c[b\times \{\beta \}]=\beta .$ To see this works, let *Y* be a subset of $\lambda ^+$ of full size, and let $\tau <\lambda ^+$ be any prescribed color, and we shall show that $\tau \in c``[Y]^2.$

As $|Y|=\lambda ^+,$ we may find a large enough $\epsilon <\lambda ^+$ such that $|Y\cap \epsilon |=\lambda$ and then find an $\alpha <\lambda ^+$ such that $Y\cap \epsilon =a_\alpha .$ Pick $\beta \in Y$ above $\max \{\epsilon ,\alpha ,\tau \}.$ Evidently, $a_\alpha \in [\beta ]^\lambda ,$ so we may pick $b\in {\mathcal {B}}_\beta$ such that $b\subseteq a_\alpha .$ Altogether, b⊆Y and $\tau \in \beta =c[b\times \{\beta \}]\subseteq c``[Y]^2.$ □

### Remark 2.2

A few remarks are in order here:

1. The proof of Theorem 2.1 is a template of a proof of anti-Ramsey consequences of the $\textsf {GCH }.$ See, e.g., [36, Theorem 9] and [29, Theorem 2] for elaborations on this argument.
2. It is not hard to see that Theorem 2.1 (and its successors) remain valid after weakening the hypothesis of $2^\lambda =\lambda ^+$ to the stick principle  In the special case of λ regular, stronger consequences may be derived—see [50, Theorem 6.3].
3. In [67, pp. 290–291], Todorčević shows that the special case of $\lambda =\aleph _0$ of Theorem 2.1 follows from a classical theorem of Sierpiński [64] asserting that the continuum hypothesis gives rise to a sequence $\langle f_n\mid n<\omega \rangle$ of functions from $\aleph _1$ to $\aleph _1$ such that for every uncountable $Y\subseteq \aleph _1,$ for all but finitely many n<ω, $f_n[Y]=\aleph _1.$ This was extended to an optimal extent in [31, Theorem 3.1].
4. A simple stretching argument shows that $\lambda ^+\nrightarrow [\lambda ^+]^2_\theta$ with $\theta =\lambda ^+$ follows from $\lambda ^+\nrightarrow [\lambda ^+]^2_\theta$ with merely $\theta =\lambda .$ This was extended in [32, §3].
5. Another easy stretching argument shows that if $\lambda \nrightarrow [\lambda ]^{<\omega }_\lambda$ then $\lambda ^+\nrightarrow [\lambda ^+]^{<\omega }_{\lambda ^+},$ and if $\kappa \nrightarrow [\kappa ]^{<\omega }_\theta$ for cofinally many $\theta <\kappa ,$ then $\kappa \nrightarrow [\kappa ]^{<\omega }_\kappa .$ See [10] and [28] for proofs and further pump-up results.

The upcoming Fact 2.4 reduces the arithmetic hypothesis of Theorem 2.1 to a postulation coming from *pcf* theory, as follows. First, given an infinite cardinal λ and a subset $\Theta \subseteq {{\,\textrm{Reg}\,}}(\lambda ),$ define an ordering $<^*$ over $\prod \Theta$ by letting $f<^*g$ iff $\sup \{ \theta \in \Theta \mid f(\theta )\ge g(\theta )\}<\sup (\Theta ).$ Then, let

- ${\mathfrak {b}}(\Theta )$ denote the least size of unbounded family in $(\prod \Theta ,<^*),$ and let
- ${\mathfrak {d}}(\Theta )$ denote the least size of a cofinal family in $(\prod \Theta ,<^*).$

### Fact 2.3

(Shelah, [57, Theorem 1.5 and Claim 2.1]) Suppose that λ is a singular cardinal. Then : 

1. There exists a cofinal subset $\Theta \subseteq {{\,\textrm{Reg}\,}}(\lambda )$ such that ${\mathfrak {d}}(\Theta )=\lambda ^+$ (and hence also ${\mathfrak {b}}(\Theta )=\lambda ^+);$
2. If ${{\,\textrm{cf}\,}}(\lambda )>\omega ,$ then the above Θ may be taken to be $\{\mu ^+\mid \mu \in C\}$ for some sparse enough club *C* in λ.

### Fact 2.4

(Todorčević, [67, pp. 289]; see [3, Theorem 4.11] for a proof) Suppose that λ is a singular cardinal. If there exists a cofinal subset $\Theta \subseteq {{\,\textrm{Reg}\,}}(\lambda )$ such that ${\mathfrak {d}}(\Theta )=\lambda ^+$ and $\theta \nrightarrow [\theta ]^2_\theta$ holds for every $\theta \in \Theta ,$ then $\lambda ^+\nrightarrow [\lambda ^+]^2_{\lambda ^+}$ holds.^1^

Recall that by the main result of [67], $\theta \nrightarrow [\theta ]^2_\theta$ holds whenever θ is a successor of a regular cardinal, thus we arrive at the following informative conclusion.

### Corollary 2.5

1. If λ is a singular cardinal satisfying $\lambda ^+\rightarrow [\lambda ^+]^2_{\lambda ^+},$ then λ is the limit of (weakly) inaccessibles; 
2. If λ is the first cardinal to satisfy $\lambda ^+\rightarrow [\lambda ^+]^2_{\lambda ^+},$ then λ is a singular cardinal of countable cofinality.

Question 6.7 of the recent paper [19] is equivalent to asking whether hypothesis of $\mathfrak {d}(\Theta )=\lambda ^+$ of Fact 2.4 may be relaxed to $\mathfrak {b}(\Theta ) =\lambda ^+.$ We answer this question in the affirmative.

### Proposition 2.6

Suppose that λ is a singular cardinal. If there exists a cofinal subset $\Theta \subseteq {{\,\textrm{Reg}\,}}(\lambda )$ such that ${\mathfrak {b}}(\Theta )=\lambda ^+$ and $\theta \nrightarrow [\theta ]^2_\theta$ holds for every $\theta \in \Theta ,$ then $\lambda ^+\nrightarrow [\lambda ^+]^2_{\lambda ^+}$ holds.

### Proof

Suppose that Θ is a cofinal subset of ${{\,\textrm{Reg}\,}}(\lambda )$ such that ${\mathfrak {b}}(\Theta )=\lambda ^+$ and $\theta \nrightarrow [\theta ]^2_\theta$ holds for every $\theta \in \Theta .$ By possibly passing to a cofinal subset, we may assume that ${{\,\textrm{otp}\,}}(\Theta )={{\,\textrm{cf}\,}}(\lambda ),$ and let $\langle \lambda _i\mid i<{{\,\textrm{cf}\,}}(\lambda )\rangle$ be the increasing enumeration of Θ. For each $i<{{\,\textrm{cf}\,}}(\lambda ),$ let $c_i:[\lambda _i]^2 \rightarrow \lambda _i$ be a witness for $\lambda _i \nrightarrow [\lambda _i]^2_{\lambda _i}.$

Using $\mathfrak {b}(\Theta )=\lambda ^+,$ we may fix an unbounded sequence $\vec {f}=\langle f_\alpha \mid \alpha <\lambda ^+\rangle$ of functions in $\prod \Theta$ satisfying, in addition, that $f_\alpha <^* f_\beta$ for all $\alpha<\beta <\lambda ^+.$ This buys us the following standard feature.

### Claim 2.6.1

For every subset $Z\subseteq \lambda ^+$ of full size,  the following set

$$\begin{aligned} I_Z:=\{ i<{{\,\textrm{cf}\,}}(\lambda )\mid \sup \{ \epsilon <\lambda _i\mid \sup \{\beta \in Z\mid f_\beta (i)=\epsilon \}=\lambda ^+\}=\lambda _i\} \end{aligned}$$

is cofinal in ${{\,\textrm{cf}\,}}(\lambda ).$

### Proof

Let $Z\subseteq \lambda ^+$ of full size be given. Fix a function $g\in \prod _{i<{{\,\textrm{cf}\,}}(\lambda )}\lambda _i$ satisfying that for every $i\in {{\,\textrm{cf}\,}}(\lambda )\setminus I_Z$:

$$\begin{aligned} g(i):=\sup \{ \epsilon <\lambda _i\mid \sup \{\beta \in Z\mid f_\beta (i)=\epsilon \}=\lambda ^+\}+1. \end{aligned}$$

As $\vec {f}$ is unbounded, there exists some $\alpha \in \lambda ^+$ such that $\{ i<{{\,\textrm{cf}\,}}(\lambda )\mid f_\alpha (i)\ge g(i)\}$ is cofinal in ${{\,\textrm{cf}\,}}(\lambda ).$ Recalling that $f_\alpha <^* f_\beta$ for all $\alpha<\beta <\lambda ^+,$ we infer the existence of a final segment *I* of $\{ i<{{\,\textrm{cf}\,}}(\lambda )\mid f_\alpha (i)\ge g(i)\}$ such that, for cofinally many $\beta \in Z,$

$$\begin{aligned} I\subseteq \{ i<{{\,\textrm{cf}\,}}(\lambda )\mid f_\beta (i)\ge g(i)\}. \end{aligned}$$

By the pigeonhole principle, $I\subseteq I_Z.$ In particular, $I_Z$ is cofinal in ${{\,\textrm{cf}\,}}(\lambda ).$ □

Next, by [15, Theorem 1], we may fix a map $d:[\lambda ^+]^2 \rightarrow [\lambda ^+]^2\times {{\,\textrm{cf}\,}}(\lambda )$ satisfying that for every $Y \subseteq \lambda ^+$ of full size, there is a stationary $Z \subseteq \lambda ^+$ such that $d``[Y]^2$ covers $[Z]^2\times {{\,\textrm{cf}\,}}(\lambda ).$ Recall that by a stretching argument, it suffices to prove that $\lambda ^+\nrightarrow [\lambda ^+]^2_\lambda$ holds. To this end, we define a coloring $c:[\lambda ^+]^2 \rightarrow \lambda$ by letting

$$\begin{aligned} c(\alpha ,\beta ):=c_i (f_\gamma (i),f_\delta (i)) \end{aligned}$$

whenever $$d(\alpha ,\beta )=(\gamma ,\delta ,i).$$

To see this works, suppose that we are given $Y\subseteq \lambda ^+$ of full size, and a prescribed color $\tau <\lambda .$ Pick $Z \subseteq \lambda ^+$ of full size such that $d``[Y]^2$ covers $[Z]^2\times {{\,\textrm{cf}\,}}(\lambda ).$ Pick a large enough $i\in I_Z$ such that $\tau <\lambda _i.$ Since $E_i:=\{ \epsilon <\lambda _i\mid \sup \{\beta \in Z\mid f_\beta (i)=\epsilon \}=\lambda ^+\}$ is cofinal in $\lambda _i,$ we may find a pair $\epsilon <\epsilon '$ of ordinals in $E_i$ such that $c_i(\epsilon ,\epsilon ')=\tau .$ Pick $\gamma \in Z$ such that $f_\gamma (i)=\epsilon$ and then pick $\delta \in Z$ above γ such that $f_\delta (i)=\epsilon '.$ Let $\alpha <\beta$ be a pair of ordinals in *Y* such that $d(\alpha ,\beta )=(\gamma ,\delta ,i).$ Then

$$\begin{aligned} c(\alpha ,\beta )=c_i(f_\gamma (i),f_\delta (i))=c_i(\epsilon ,\epsilon ')=\tau , \end{aligned}$$

as sought. □

Recall that the *singular cardinals hypothesis* ($\textsf {SCH }$) is the assertion that $\textsf {SCH }_\lambda$ holds for every singular cardinal λ, where $\textsf {SCH }_\lambda$ means that if $2^{{{\,\textrm{cf}\,}}(\lambda )}<\lambda ,$ then $2^\lambda =\lambda ^+.$ Motivated by Proposition 2.6, we formulate the following particular failure of the $\textsf {SCH }$:

### Definition 2.7

For a property φ of a cardinal (such as being an inaccessible) and a singular cardinal λ, the φ *failure of* $\textsf {SCH }_\lambda$ asserts that for every cofinal $\Theta \subseteq {{\,\textrm{Reg}\,}}(\lambda )$ with $\mathfrak {b}(\Theta )=\lambda ^+,$ it is the case that co-boundedly many $\theta \in \Theta$ have property φ.

### Problem 1

Is the weakly compact failure of $\textsf {SCH }_\lambda$ consistent (from a large cardinals hypothesis)?

Note that by Solovay’s theorem [65, Theorem 1], the strongly compact failure of $\textsf {SCH }_\lambda$ is inconsistent. D. Adolf informed us that the consistency of the weakly compact failure of $\textsf {SCH }_\lambda$ would require at least a Woodin cardinal.

Ben-Neria suggested that an affirmative answer to Problem 1 may be obtained using the forcing of Merimovich from [35]. Specifically, suppose j:V→M is a *V*-definable nontrivial elementary embedding with critical point λ. Denote $\mu := j(\lambda )$ and $\kappa := j(\mu )$ and further assume  and $V_{\kappa } \subseteq M.$ Let $\vec {E}$ be the extender derived from *j* with measures $\vec {E}(d)$ for $d \in [\kappa ]^{<\mu },$ and finally let ${\mathbb {P}}_{\vec {E}}$ be the associated extender-based Prikry forcing of [35]. Merimovich proved that this forcing changes the cofinality of λ to ω, does not add bounded subsets of λ, collapses the cardinals in the interval $(\lambda ,\mu )$ and only those, and adds κ-many ω-sequences to λ. Ben-Neria claims that $V^{{\mathbb {P}}_{\vec {E}}}$ witnesses the measurable failure of $\textsf {SCH }_\lambda .$

## Compactness and incompactness

We mentioned in the introduction Todorčević’s theorem [67] that the existence of a nonreflecting stationary subset of κ implies that $\kappa \nrightarrow [\kappa ]^2_\kappa$ holds. This was later extended in [43] to get a strong block-wise version of $\kappa \nrightarrow [\kappa ]^2_\kappa .$ Extending Todorčević’s theorem in a different direction, in [26], it was proved that an inaccessible cardinal κ admitting a stationary set that merely does not reflect at regulars already satisfies $\kappa \nrightarrow [\kappa ]^2_\kappa .$ As for successors of singulars, we have the following:

### Fact 3.1

(Eisworth, [14]) Suppose that λ is a singular cardinal such that $\lambda ^+\rightarrow [\lambda ^+]^2_{\lambda ^+}$ holds. Then every family ${\mathcal {S}}$ of less than ${{\,\textrm{cf}\,}}(\lambda )$-many stationary subsets of $\lambda ^+$ reflects simultaneously.^2^

The combination of the preceding fact with the results of the previous section prompts the study of models in which there exists a singular cardinal λ of countable cofinality such that $\textsf {SCH }_\lambda$ fails and simultaneous reflection of stationary subsets of $\lambda ^+$ holds. The existence of such a model was demonstrated only recently. In [40], Poveda et al. obtained such a model using iterated Prikry-type forcing, continuing the work of Sharon [54]. In [2], Ben-Neria et al. obtained such a model using iterated ultrapowers. A third model was constructed by Gitik in [20]. A possible explanation of the difficulty in getting such a model is in the fact that the failure of $\textsf {SCH }$ implies the failure of reflection of *two-cardinal* stationary sets [17, 51, 61, 69].

The failure of $\textsf {SCH }_\lambda$ has combinatorial consequences, as well. For instance, it implies the existence of a so-called *better scale* at λ which the proof of [7, Theorem 4.1] shows to imply the combinatorial principle ${{\,\textrm{ADS}\,}}_\lambda .$ It is reasonable to expect that the weakly compact failure of $\textsf {SCH }_\lambda$ would have nicer combinatorial consequences.

### Problem 2

Find combinatorial consequences of the weakly compact failure of $\textsf {SCH }_\lambda .$

We continue with two more facts connecting incompactness to strong colorings.

### Fact 3.2

(Jensen, [27, Lemma 6.6]; Shore [62, Lemma 1]) If there is a κ-Souslin tree,  then $\kappa \nrightarrow [\kappa ]^2_\kappa$ holds.

### Fact 3.3

[26, §7] If $\square (\kappa ,{<}\omega )$ holds,  then so does $\kappa \nrightarrow [\kappa ]^2_\kappa .$.^3^

Recalling that $\square (\kappa ,{<}\kappa )$ is equivalent to the existence of a κ-Aronszajn tree, we see that the hypotheses of the last two facts have to do with particular forms of κ-Aronszajn trees. So, we ask:

### Problem 3

Suppose that λ is a singular cardinal and there exists a $\lambda ^+$-Aronszajn tree. Does $\lambda ^+ \nrightarrow [\lambda ^+]^2_\lambda$ hold?

Taking into account all of the results discussed so far, we arrive at the following difficult problem:

### Problem 4

Modulo a large cardinals hypothesis, is the conjunction of the following consistent for some singular cardinal λ?

- Weakly compact failure of $\textsf {SCH }_\lambda ;$
- ${{\,\textrm{TP}\,}}(\lambda ^+),$ i.e., there are no $\lambda ^+$-Aronszajn trees;
- Every finite family of stationary subsets of $\lambda ^+$ reflects simultaneously.

The first model to satisfy the (usual) failure of $\textsf {SCH }_\lambda$ together with ${{\,\textrm{TP}\,}}(\lambda ^+)$ holding was constructed by Neeman in [37]. The state of the art with respect to this research direction may be found at [8].

We end this section by pointing out that the combination of Fact 3.2 and Problem 3 gives rise to the following problem (a variation of [52, Problem 1] that was studied in [6]):

### Problem 5

Suppose that λ is a singular cardinal and there exists a $\lambda ^+$-Aronszajn tree. Does there exist a $\lambda ^+$-Souslin tree?

We find the preceding problem to be of interest even in the context of $\textsf {GCH }.$ The point is that the main result of [5] shows that in this context, singularizations of a regular cardinal λ tend to do introduce $\lambda ^+$-Souslin trees. Finally, in view of the fact that $\square (\lambda ^+,\lambda )$ is equivalent to the existence of a $\lambda ^+$-Aronszajn tree, the following variation of Problem 5 emerges:

### Problem 6

Suppose that λ is a singular cardinal and $\square (\lambda ^+,{<}\lambda )$ and $\textsf {GCH }$ both hold. Does there exist a $\lambda ^+$-Souslin tree?

An affirmative answer is known once replacing $\square (\lambda ^+,{<}\lambda )$ with the stronger hypothesis $\square (\lambda ^+)$ [46], or replacing ‘singular’ by ‘regular uncountable’ [47]. By [4, Corollary 2.24], the hypothesis of Problem 6 is sufficient for the construction of a *distributive* $\lambda ^+$-Aronszajn tree.

## Reductions and approximations

Recall that by Remark 2.2(4), $\lambda ^+\nrightarrow [\lambda ^+]^2_\theta$ with $\theta =\lambda ^+$ follows from $\lambda ^+\nrightarrow [\lambda ^+]^2_\theta$ with merely $\theta =\lambda .$ The next fact provides a further reduction.

### Fact 4.1

(Eisworth, [15]) Suppose that λ is a singular cardinal. If $\lambda ^+ \nrightarrow [\lambda ^+]^2_\theta$ holds for arbitrarily large $\theta <\lambda ,$ then $\lambda ^+ \nrightarrow [\lambda ^+]^2_{\lambda }$ holds.

As of today, the largest number of colors known to be attainable is rather small:

### Fact 4.2

(Shelah, [57, Conclusion 4.1]) For every singular cardinal λ, $\lambda ^+ \nrightarrow [\lambda ^+]^2_{{{\,\textrm{cf}\,}}(\lambda )}$ holds.

In view of the last two facts, we ask:

### Problem 7

Suppose that λ is a singular cardinal. Does $\lambda ^+ \nrightarrow [\lambda ^+]^2_{{{\,\textrm{cf}\,}}(\lambda )^+}$ hold?

Note that $\lambda ^+ \nrightarrow [\lambda ^+]^2_{{{\,\textrm{cf}\,}}(\lambda )^+}$ is equivalent to the syntactically-weaker principle ${{\,\textrm{U}\,}}(\lambda ^+,2,{{\,\textrm{cf}\,}}(\lambda )^+,2)$ of [33, Definition 1.2].^4^ We do not know whether this equivalence remains true replacing ${{\,\textrm{cf}\,}}(\lambda )^+$ by an arbitrary regular cardinal $\theta <\lambda .$

### Definition 4.3

Given a coloring $c:[\lambda ^+]^2 \rightarrow \theta$ and a cardinal $\mu \le \lambda ,$ consider the following notion of forcing

$$\begin{aligned} {\mathbb {P}}_{c,\mu }:=(\{ x \in [\lambda ^+]^{<\mu }\mid c\mathbin \upharpoonright [x]^2{\text { is constant}}\},\supseteq ) \end{aligned}$$

for adding a large *c*-homogeneous set, so that *c* will not witness $\lambda ^+ \nrightarrow [\lambda ^+]^2_\theta .$

### Fact 4.4

[42, Theorem 1] Suppose that λ is a singular cardinal and $\theta \le \lambda ^+$ is any cardinal. If $\lambda ^+ \nrightarrow [\lambda ^+]^2_\theta$ holds,  then it may be witnessed by a coloring $c:[\lambda ^+]^2 \rightarrow \theta$ for which ${\mathbb {P}}_{c,\mu }$ has the $\lambda ^+$-cc for every cardinal μ such that $\lambda ^{<\mu }=\lambda .$

This ensures that cardinals above λ will not be collapsed. But what about the ones from below? Here, we have some bad news indicating that ${\mathbb {P}}_{c,\mu }$ is not the right poset for this task.

### Fact 4.5

[50, Proposition 2.10 and 2.12] Suppose that $c:[\lambda ^+]^2 \rightarrow 2$ is a coloring for some singular cardinal λ. Then : 

- ${\mathbb {P}}_{c,\lambda }$ has an antichain of size $\lambda ^+$ consisting of pairwise disjoint sets; 
- If λ is the limit of strongly compacts,  then the above is true already for ${\mathbb {P}}_{c,{{\,\textrm{cf}\,}}(\lambda )^+}.$

### Problem 8

Given a coloring $c:[\lambda ^+]^2 \rightarrow \theta$ witnessing $\lambda ^+ \nrightarrow [\lambda ^+]^2_\theta ,$ is there a cofinality-preserving notion of forcing for ‘killing’ *c*? Identify additional features of *c* that would enable an affirmative answer.

To demonstrate the difficulty in solving Problem 8, examine the very proof of Fact 4.2 and see what it takes to ‘kill’ the particular coloring constructed there.

The next reduction tells us that we may focus our attention on sets thicker than just cofinal.

### Fact 4.6

[42, Theorem 2] Suppose that λ is a singular cardinal. If there are a cardinal $\mu <\lambda$ and a coloring $c:[\lambda ^+]^2 \rightarrow \theta$ such that $c``[S]^2=\theta$ for every *stationary* $S \subseteq E^{\lambda ^+}_{>\mu },$ then $\lambda ^+ \nrightarrow [\lambda ^+]^2_\theta$ holds.

### Problem 9

Identify interesting ideals *J* over $\lambda ^+$ for which $\textsf {ZFC }$ proves the existence of a coloring $c:[\lambda ^+]^2 \rightarrow \lambda$ satisfying $c``[B]^2=\lambda$ for every $B\in J^+.$

The next reduction tells us that instead of implementing prescribed colors as $c(\alpha ,\beta )$ for some pair $\alpha <\beta$ of ordinals from a given large set *Y*,  it often suffices to do so as c(i,β) where only β is required to come from *Y*.

### Fact 4.7

[23, Lemma 8.9(2)] Suppose a singular cardinal λ is a strong limit or satisfies that $\aleph _\lambda >\lambda .$ If there exists a coloring $c:\lambda \times \lambda ^+ \rightarrow \lambda$ such that for every $Y \subseteq \lambda ^+$ of full size, there is i<λ with $c[\{i\}\times Y]=\lambda ,$ then $\lambda ^+ \nrightarrow [\lambda ^+]^2_\lambda$ holds.

Unfortunately, in [25] it was shown that in the model of [22] there is no such coloring $c:\lambda \times \lambda ^+\rightarrow \lambda .$ But we do get two approximations of such a coloring in $\textsf {ZFC }$:

### Fact 4.8

[25, Theorem 6.9 and Corollary 6.12] Suppose that λ is a singular cardinal. Then : 

1. There is a coloring $c:\lambda \times \lambda ^+ \rightarrow \lambda$ such that for every $Y \subseteq \lambda ^+$ of full size,  there is i<λ with ${{\,\textrm{otp}\,}}(c[\{i\}\times Y])=\lambda ;$
2. For every cardinal $\theta <\lambda ,$ there is a coloring $c:\lambda \times \lambda ^+ \rightarrow \theta$ such that for every $Y \subseteq \lambda ^+$ of full size,  there is i<λ with $c[\{i\}\times Y]=\theta .$

To appreciate the fact that Clause (1) holds in $\textsf {ZFC }$ for λ singular, note that the same assertion for λ regular is equivalent to $\mathfrak {b}_\lambda =\lambda ^+$ (see [25, Lemma 6.1]).

## Connections to algebra

Jónsson cardinals are known to have tight connections to problems in algebra, and in particular, group theory. First, consider the following partition relation.

### Definition 5.1

For an Abelian group *G*,  $G\nrightarrow [\lambda ]^{{{\,\textrm{FS}\,}}}_\theta$ asserts the existence of a coloring $c:G\rightarrow \theta$ such that $c\mathbin \upharpoonright {{\,\textrm{FS}\,}}(Y)$ is surjective for every Y⊆G of size λ.^5^

Hindman’s celebrated theorem yields that $G\rightarrow [\omega ]^{{{\,\textrm{FS}\,}}}_n$ holds (i.e., $G\nrightarrow [\omega ]^{{{\,\textrm{FS}\,}}}_n$ fails) for every positive integer *n* and every infinite Abelian group *G*.

Applying the relation of Definition 5.1 to the Abelian group $G:=([\kappa ]^{<\omega },\bigtriangleup )$ readily implies $\kappa \nrightarrow [\lambda ]^{<\omega }_\theta .$ But more is true, namely, by [18, Corollary 2.8], $\kappa \nrightarrow [\lambda ]^{<\omega }_\theta$ holds iff $G\nrightarrow [\lambda ]^{{{\,\textrm{FS}\,}}}_\theta$ holds for every Abelian group *G* of size κ. The special case $\lambda =\theta =\kappa$ of this result provides the following characterization:

### Corollary 5.2

κ is not Jónsson iff $G\nrightarrow [\kappa ]^{{{\,\textrm{FS}\,}}}_\kappa$ holds for every Abelian group *G* of size κ.

A second characterization centers around non-Abelian groups. A *Jónsson group* is a group *G* such that none of its proper subgroups have size |*G*|. By [53], the only infinite Abelian Jónsson groups are the Prüfer *p*-groups, in particular, uncountable Jónsson groups are non-Abelian. But do they even exist?

### Fact 5.3

(Corson, [9]) κ is not Jónsson iff there exist a Jónsson group of size κ.

The first uncountable Jónsson group was constructed by Shelah in [55], answering a question of Kurosh. In the same paper the following strengthening of a Jónsson group was considered, and later became known as a *Shelah group*.

### Definition 5.4

A *Shelah group* is a group *G* for which there exists a positive integer *n* such that for every Y⊆G of full size, $Y^n=G,$ that is, every element of *G* may be written as a group word of length exactly *n* in the elements of *Y*.

In [55], Shelah proved that $2^\lambda =\lambda ^+$ entails the existence of a Shelah group of size $\lambda ^+.$ In [38], it was shown that the arithmetic hypothesis is redundant in the case that λ is regular. Back to successors of singulars, we ask:

### Problem 10

Suppose that λ is a singular cardinal such that $\lambda ^+$ is not Jónsson. Does there exist a Shelah group of size $\lambda ^+?$

It can be shown that the existence of a Shelah group of size $\lambda ^+$ with a corresponding integer *n* implies $\lambda ^+\nrightarrow [\lambda ^+]^{n+1}_{\lambda ^+}.$ This raises the question whether the following pump-up is possible:

### Problem 11

Suppose that λ is a singular cardinal such that $\lambda ^+$ is not Jónsson. Does there exist a large enough positive integer *n* such that $\lambda ^+\nrightarrow [\lambda ^+]^n_{\lambda ^+}$ hold?

A problem of a somewhat similar flavor is studied in [66].

## Club guessing

For a set of ordinals *S*,  a *C*-*sequence over* *S* is a sequence $\vec {C}=\langle C_\delta \mid \delta \in S\rangle$ satisfying that for every $\delta \in S,$ $C_\delta$ is a closed subset of δ with $\sup (C_\delta )=\sup (\delta ).$

### Definition 6.1

(*Shelah*) A *C*-sequence $\vec {C}=\langle C_\delta \mid \delta \in S\rangle$ over a stationary subset $S\subseteq \kappa$ is said to *guess clubs* iff for every club $D\subseteq \kappa ,$ there is some $\delta \in S$ with $C_\delta \subseteq D.$

By [58, §1], for every stationary $S\subseteq \kappa$ such that $\sup \{ {{\,\textrm{cf}\,}}(\delta )^+\mid \delta \in S\}<\kappa ,$ there exists a *C*-sequence over *S* that guesses clubs. We propose the following terminology:

### Definition 6.2

A *C*-sequence $\vec {C}=\langle C_\delta \mid \delta \in S\rangle$ is *uninhibited* iff for club many $\delta \in S,$ for every $\mu \in {{\,\textrm{Reg}\,}}(\delta ),$ $\sup ({{\,\textrm{nacc}\,}}(C_\delta )\cap E^{\delta }_{\ge \mu })=\delta .$

### Definition 6.3

Given a *C*-sequence $\vec {C}=\langle C_\delta \mid \delta \in S\rangle$ over some stationary $S\subseteq \kappa ,$ consider the following corresponding ideal:

$$\begin{aligned} J(\vec {C}):=\left\{ A \subseteq \kappa \Bigg |\begin{array}{c}\exists {\text {club }}D \subseteq \kappa \forall \delta \in S\cap D\exists \mu \in {{\,\textrm{Reg}\,}}(\delta )\\ \sup ({{\,\textrm{nacc}\,}}(C_\delta )\cap E^{\delta }_{\ge \mu }\cap D\cap A)<\delta \end{array}\right\} . \end{aligned}$$

By [58, Claim 2.4], for every regular uncountable cardinal λ, there exists a *C*-sequence $\vec {C}$ over $E^{\lambda ^+}_{\lambda }$ for which $J(\vec {C})$ is proper. By [16, Theorem 2], for every singular cardinal λ of uncountable cofinality, there moreover exists an uninhibited club-guessing *C*-sequence $\vec {C}$ over $E^{\lambda ^+}_{{{\,\textrm{cf}\,}}(\lambda )}.$ The following is Question 2.4 of [16] and is still open:

### Problem 12

Suppose that λ is a singular cardinal of countable cofinality. Must there exist an uninhibited club-guessing *C*-sequence over $E^{\lambda ^+}_{{{\,\textrm{cf}\,}}(\lambda )}$?

In Remark 2.2(5), we mentioned a couple of easy pump-up results. In [58, Lemma 1.9], Shelah proved a deep pump-up theorem asserting that for every singular cardinal λ, if there exists a *C*-sequence $\vec {C}$ over $E^{\lambda ^+}_{{{\,\textrm{cf}\,}}(\lambda )}$ for which there exists a $J(\vec {C})$-positive subset of $\{ \beta <\lambda ^+\mid {{\,\textrm{cf}\,}}(\beta )$ is not Jónsson}, then $\lambda ^+$ is not Jónsson. Coming back to the context of coloring pairs, we have the following result concerning the ideal of Definition 6.3:

### Fact 6.4

(Eisworth, [12]) Suppose that λ is a singular cardinal,  $\vec {C}=\langle C_\delta \mid \delta \in E^{\lambda ^+}_{{{\,\textrm{cf}\,}}(\lambda )}\rangle$ is a *C*-sequence such that ${{\,\textrm{otp}\,}}(C_\delta )<\lambda$ for all $\delta \in E^{\lambda ^+}_{{{\,\textrm{cf}\,}}(\lambda )}.$ Whenever there are θ-many pairwise disjoint $J(\vec {C})$-positive sets,  $\lambda ^+\nrightarrow [\lambda ^+]^2_\theta$ holds.^6^

Note that for a singular λ and a $\vec {C}$ over $E^{\lambda ^+}_{{{\,\textrm{cf}\,}}(\lambda )},$ for every regular cardinal θ distinct from ${{\,\textrm{cf}\,}}(\lambda ),$ the ideal $J(\vec {C})$ is θ-indecomposable, i.e., it is closed under taking ⊆-increasing unions of length θ. Eisworth’s proof of Fact 3.1 builds on the preceding together with [13, §2] that studies the conjunction of θ-indecomposability and weak θ-saturation.^7^ Further non-weak-saturation results for indecomposable ideals were recently obtained in [26].

### Corollary 6.5

(Eisworth) Suppose that λ is a singular cardinal,  and $\vec {C}=\langle C_\delta \mid \delta \in E^{\lambda ^+}_{{{\,\textrm{cf}\,}}(\lambda )}\rangle$ is a club guessing *C*-sequence such that ${{\,\textrm{otp}\,}}(C_\delta )=\lambda$ for all $\delta \in E^{\lambda ^+}_{{{\,\textrm{cf}\,}}(\lambda )}.$ Then $\lambda ^+ \nrightarrow [\lambda ^+]^2_{\lambda }$ holds.

### Proof

Using a general partition theorem for club guessing [24, Proposition 4.24], we may fix a partition $\langle S_\tau \mid \tau <\lambda \rangle$ of $E^{\lambda ^+}_{{{\,\textrm{cf}\,}}(\lambda )}$ such that $\vec {C}\mathbin \upharpoonright S_\tau$ guesses clubs for each $\tau < \lambda .$ By Corollary 2.5(1), we may assume that $\lambda =\aleph _\lambda ,$ hence, we may find a pairwise disjoint sequence $\vec {K}=\langle K_\tau \mid \tau <\lambda \rangle$ of cofinal subsets of ${{\,\textrm{Reg}\,}}(\lambda )$ of order-type ${{\,\textrm{cf}\,}}(\lambda ).$ For every $\tau <\lambda ,$ set $B_\tau :=\{ \beta <\lambda ^+\mid {{\,\textrm{cf}\,}}(\beta )\in K_\tau \},$ and note that $\vec {B}=\langle B_\tau \mid \tau <\lambda \rangle$ is a sequence of pairwise disjoint stationary subsets of $\lambda ^+.$

Next, for all $\tau <\lambda$ and $\delta \in S_\tau ,$ let $D_\delta$ be the closure below δ of the following set

$$\begin{aligned} \{\gamma \in C_\delta \mid {{\,\textrm{otp}\,}}(C_\delta \cap \gamma )\in K_\tau \}, \end{aligned}$$

so that $D_\delta$ is a subclub of $C_\delta$ of order-type ${{\,\textrm{cf}\,}}(\lambda ).$ In particular, $\vec {D}:=\langle D_\delta \mid \delta \in E^{\lambda ^+}_{{{\,\textrm{cf}\,}}(\lambda )}\rangle$ is an uninhibited club-guessing *C*-sequence such that ${{\,\textrm{otp}\,}}(D_\delta )<\lambda$ for all $\delta \in E^{\lambda ^+}_{{{\,\textrm{cf}\,}}(\lambda )}.$ A moment’s reflection makes it clear that, for every $\tau <\lambda ,$ $B_\tau$ is $J(\vec {D}\mathbin \upharpoonright S_\tau )$-positive. In particular, $\vec {B}$ consists of λ-many pairwise disjoint $J(\vec {D})$-positive sets. By Fact 6.4, then, $\lambda ^+\nrightarrow [\lambda ^+]^2_\lambda$ holds. □

The preceding motivates the following stronger form of Problem 12:

### Problem 13

Suppose that λ is a singular cardinal. Is there a club guessing *C*-sequence $\vec {C}=\langle C_\delta \mid \delta \in E^{\lambda ^+}_{{{\,\textrm{cf}\,}}(\lambda )}\rangle$ such that ${{\,\textrm{otp}\,}}(C_\delta )=\lambda$ for all $\delta \in E^{\lambda ^+}_{{{\,\textrm{cf}\,}}(\lambda )}$?

To compare, a negative answer is known for λ regular, as Abraham and Shelah proved [1] it is consistent for a regular cardinal λ to have $\lambda ^{++}$ many clubs in $\lambda ^+$ such that the intersection of any $\lambda ^+$ many of them has size <λ.

Problem 13 bears similarity to questions concerning the failure of diamond at successor of singulars; results in this direction may be found in [21, 56, 60].

An affirmative answer to Problem 13 (or Problem 12) under the additional assumption of $\square ^*_\lambda$ is of interest, as well.

We conclude this paper by repeating Question 2 from [44]:

### Problem 14

Suppose that λ is a singular cardinal. Assuming $\square _\lambda$ holds, may it be witnessed by *C*-sequence $\vec {C}=\langle C_\delta \mid \delta <\lambda ^+\rangle$ with the property that for every club $D\subseteq \lambda ^+,$ there exists some $\delta \in {{\,\textrm{acc}\,}}(\lambda ^+)$ such that ${{\,\textrm{otp}\,}}(C_\delta \cap D)=\lambda$?

By the main result of [45], an affirmative answer to the preceding holds assuming $2^\lambda =\lambda ^+.$

## Footnotes

1 An analogous pump-up fact holds true for Jónsson-ness—see [57, Conclusion 4.6(γ)].

2 This means that there exists an ordinal $\delta <\lambda ^+$ of uncountable cofinality such that $S\cap \delta$ is stationary in δ for every $S\in {\mathcal {S}}.$

3 The definition of square principles may be found at [4, Definition 1.16] The stronger hypothesis $\square (\kappa )$ yields a stronger block-wise version of $\kappa \nrightarrow [\kappa ]^2_\kappa$ [49, Theorem ${\textrm{A}}^{\prime }$].

4 A sufficient condition for ${{\,\textrm{U}\,}}(\lambda ^+,2,{{\,\textrm{cf}\,}}(\lambda )^+,2)$ to hold may be found at [34, Lemma 3.7].

5 Here, ${{\,\textrm{FS}\,}}(Y)$ stands for the set of all finite sums $y_1+\cdots +y_n$ of distinct elements of *Y*.

6 Compare with [59, Remark 2.10(2)].

7 An ideal *J* is *weakly* θ-*saturated* iff $J^+$ admits no pairwise disjoint family of size θ.

## References

[1] Abraham, U., Shelah, S.: On the intersection of closed unbounded sets. J. Symb. Log. **51**, 180–189 (1986)

[2] Ben-Neria, O., Hayut, Y., Unger, S.: Stationary reflection and the failure of the SCH. J. Symb. Log. **89**(1), 1–26 (2024)

[3] Burke, M.R., Magidor, M.: Shelah’s theory and its applications. Ann. Pure Appl. Log. **50**(3), 207–254 (1990)

[4] Brodsky, A.M., Rinot, A.: Distributive Aronszajn trees. Fund. Math. **245**(3), 217–291 (2019)

[5] Brodsky, A.M., Rinot, A.: More notions of forcing add a Souslin tree. Notre Dame J. Form. Log. **60**(3), 437–455 (2019)

[6] Brodsky, A.M., Rinot, A.: A remark on Schimmerling’s question. Order **36**(3), 525–561 (2019)

[7] Cummings, J., Foreman, M., Magidor, M.: Squares, scales and stationary reflection. J. Math. Log. **1**(1), 35–98 (2001)

[8] Cummings, J., Hayut, Y., Magidor, M., Neeman, I., Sinapova, D., Unger, S.: The tree property on long intervals of regular cardinals (2025). arXiv preprint. arXiv:2505.08118

[9] Corson, S.M.: Jónsson groups of various cardinalities. Proc. Am. Math. Soc. **150**(7), 2771–2775 (2022)

[10] Devlin, K.J.: Some weak versions of large cardinal axioms. Ann. Math. Log. 5:291–325 (1972/1973)

[11] Erdős, P., Hajnal, A., Rado, R.: Partition relations for cardinal numbers. Acta Math. Acad. Sci. Hung. **16**, 93–196 (1965)

[12] Eisworth, T.: A note on strong negative partition relations. Fund. Math. **202**(2), 97–123 (2009)

[13] Eisworth, T.: Club-guessing, stationary reflection, and coloring theorems. Ann. Pure Appl. Log. **161**(10), 1216–1243 (2010)

[14] Eisworth, T.: Simultaneous reflection and impossible ideals. J. Symb. Log. **77**(4), 1325–1338 (2012)

[15] Eisworth, T.: Getting more colors I. J. Symb. Log. **78**(1), 1–16 (2013)

[16] Eisworth, T., Shelah, S.: Successors of singular cardinals and coloring theorems. II. J. Symb. Log. **74**, 1287–1309 (2009)

[17] Fuchino, S., Rinot, A.: Openly generated Boolean algebras and the Fodor-type reflection principle. Fund. Math. **212**(3), 261–283 (2011)

[18] Fernandez-Breton, D., Rinot, A.: Strong failures of higher analogs of Hindman’s theorem. Trans. Am. Math. Soc. **369**(12), 8939–8966 (2017)

[19] Gilton, T.: PCF theory and the Tukey spectrum. Fund. Math. **265**(1), 15–33 (2024)

[20] Gitik, M.: Reflection and not SCH with overlapping extenders. Arch. Math. Log. **61**(5–6), 591–597 (2022)

[21] Gitik, M., Rinot, A.: The failure of diamond on a reflecting stationary set. Trans. Am. Math. Soc. **364**(4), 1771–1795 (2012)

[22] Garti, S., Shelah, S.: A strong polarized relation. J. Symb. Log. **77**, 766–776 (2012)

[23] Inamdar, T., Rinot, A.: Was Ulam right? I: Basic theory and subnormal ideals. Topol. Appl. **323**(C), Paper No. 108287 pp. 65 (2023)

[24] Inamdar, T., Rinot, A.: A club guessing toolbox I. Bull. Symb. Log. **30**(3), 303–361 (2024)

[25] Inamdar, T., Rinot, A.: Was Ulam right? II: Small width and general ideals. Algebra Universalis **85**(2), Paper No. 14 pp. 47 (2024)

[26] Inamdar, T., Rinot, A.: Was Ulam right? III: Indecomposable ideals (2025). Submitted April 2025. http://assafrinot.com/paper/59

[27] Jensen, R.B.: The fine structure of the constructible hierarchy. Ann. Math. Log. **4**, 229–308 (1972). [Erratum, ibid. **4**, 443 (1972). With a section by Jack Silver]

[28] Kleinberg, E.M.: Rowbottom cardinals and Jonsson cardinals are almost the same. J. Symb. Log. **38**, 423–427 (1973)

[29] Komjáth, P.: Weiss’s question. Mathematika **66**(4), 954–958 (2020)

[30] Keisler, H.J., Rowbottom, F.: Constructible sets and weakly compact cardinals. Not. Am. Math. Soc. **12**, 373 (1965)

[31] Kojman, M., Rinot, A., Steprans, J.: Ramsey theory over partitions III: strongly Luzin sets and partition relations. Proc. Am. Math. Soc. **151**(1), 369–384 (2023)

[32] Kojman, M., Rinot, A., Steprans, J.: Ramsey theory over partitions II: negative Ramsey relations and pump-up theorems. Isr. J. Math. **261**(1), 223–247 (2024)

[33] Lambie-Hanson, C., Rinot, A.: Knaster and friends I: closed colorings and precalibers. Algebra Univers. **79**(4), Art. 90, pp. 39 (2018)

[34] Lambie-Hanson, C., Rinot, A.: Knaster and friends III: subadditive colorings. J. Symb. Log. **88**(3), 1230–1280 (2023)

[35] Merimovich, C.: Supercompact extender based Prikry forcing. Arch. Math. Log. **50**(5–6), 591–602 (2011)

[36] Milliken, K.R.: Hindman’s theorem and groups. J. Comb. Theory Ser. A **25**(2), 174–180 (1978)

[37] Neeman, I.: Aronszajn trees and failure of the singular cardinal hypothesis. J. Math. Log. **9**(1), 139–157 (2009)

[38] Poor, M., Rinot, A.: A Shelah group in ZFC. Forum Math. Pi **13**, 1–28 (2025)

[39] Prikry, K.L.: Changing measurable into accessible cardinals. Diss. Math. (Rozprawy Mat.) **68**, 55 (1970)

[40] Poveda, A., Rinot, A., Sinapova, D.: Sigma-Prikry forcing II: iteration scheme. J. Math. Log. **22**(3), Paper No. 2150019 pp. 50 (2022)

[41] Ramsey, F.P.: On a problem of formal logic. Proc. Lond. Math. Soc. **30**, 264–286 (1930)

[42] Rinot, A.: Transforming rectangles into squares, with applications to strong colorings. Adv. Math. **231**(2), 1085–1099 (2012)

[43] Rinot, A.: Complicated colorings. Math. Res. Lett. **21**(6), 1367–1388 (2014)

[44] Rinot, A.: The Ostaszewski square, and homogeneous Souslin trees. Isr. J. Math. **199**(2), 975–1012 (2014)

[45] Rinot, A.: Putting a diamond inside the square. Bull. Lond. Math. Soc. **47**(3), 436–442 (2015)

[46] Rinot, A.: Higher Souslin trees and the GCH, revisited. Adv. Math. **311**(C), 510–531 (2017)

[47] Rinot, A.: Souslin trees at successors of regular cardinals. MLQ Math. Log. Q. **65**(2), 200–204 (2019)

[48] Rowbottom, F.: Some strong axioms of infinity incompatible with the axiom of constructibility. Ann. Math. Log. **3**(1), 1–44 (1971)

[49] Rinot, A., Zhang, J.: Complicated colorings, revisited. Ann. Pure Appl. Log. **174**(4), Paper No. 103243 (2023)

[50] Rinot, A., Zhang, J.: Strongest transformations. Combinatorica **43**(1), 149–185 (2023)

[51] Sakai, H.: Simple proofs of SCH from reflection principles without using better scales. Arch. Math. Log. **54**(5–6), 639–647 (2015)

[52] Schimmerling, E.: A question about Suslin trees and the weak square hierarchy. Notre Dame J. Formal Log. **46**(3), 373–374 (electronic) (2005)

[53] Scott, W.R.: Groups and cardinal numbers. Am. J. Math. **74**, 187–197 (1952)

[54] Sharon, A.: Weak squares, scales, stationary reflection and the failure of SCH. Ph.D. Thesis, Tel Aviv University, March (2005)

[55] Shelah, S.: On a problem of Kurosh, Jónsson groups, and applications. In: Word Problems, II (Conference on Decision Problems in Algebra, Oxford, 1976). Studies in Logic and the Foundations of Mathematics, vol. 95, pp. 373–394. North-Holland, Amsterdam (1980)

[56] Shelah, S.: Diamonds, uniformization. J. Symb. Log. **49**, 1022–1033 (1984)

[57] Shelah, S.: has a Jonsson algebra. In: Cardinal Arithmetic. Oxford Logic Guides, vol. 29. Oxford University Press, Oxford (1994)

[58] Shelah, S.: There are Jonsson algebras in many inaccessible cardinals. In: Cardinal Arithmetic. Oxford Logic Guides, vol. 29. Oxford University Press, Oxford (1994)

[59] Shelah, S.: More Jonsson algebras. Arch. Math. Log. **42**, 1–44 (2003)

[60] Shelah, S.: Successor of singulars: combinatorics and not collapsing cardinals in -support iterations. Isr. J. Math. **134**, 127–155 (2003)

[61] Shelah, S.: Reflection implies the SCH. Fund. Math. **198**(2), 95–111 (2008)

[62] Shore, R.A.: Square bracket partition relations in . Fund. Math. **84**(2), 101–106 (1974)

[63] Sierpiński, W.: Sur un problème de la théorie des relations. Ann. Scuola Norm. Sup. Pisa Cl. Sci. (2) **2**(3), 285–287 (1933)

[64] Sierpiński, W.: Hypothèse du continu. Monografie Matematyczne, Z Subwencji Funduszu Kultury Narodowej, Warszawa-Lwów **4**, v+192 (1934). http://eudml.org/doc/219323

[65] Solovay, R.M.: Strongly compact cardinals and the GCH. In: Proceedings of the Tarski Symposium (Proceedings of the Symposium in Pure Mathematics, vol. XXV, Univ. California, Berkeley, Calif., American Mathematical Society, Providence, RI 1971), pp. 365–372 (1974)

[66] Shelah, S., Steprāns, J.: Higher dimensional universal functions from lower dimensional ones. Preprint, March (2023). https://shelah.logic.at/papers/1241

[67] Todorčević, S.: Partitioning pairs of countable ordinals. Acta Math. **159**(3–4), 261–294 (1987)

[68] Tryba, J.: On Jónsson cardinals with uncountable cofinality. Isr. J. Math. **49**(4), 315–324 (1984)

[69] Viale, M.: The proper forcing axiom and the singular cardinal hypothesis. J. Symb. Log. **71**(2), 473–479 (2006)

